# Authors’ reply regarding “Does tattoo exposure increase the risk of cutaneous melanoma: A population based case-control study”

**DOI:** 10.1007/s10654-026-01381-7

**Published:** 2026-03-13

**Authors:** Emelie Rietz Liljedahl, Kari Nielsen, Malin Engfeldt, Anna Saxne Jöud, Christel Nielsen

**Affiliations:** 1 Division of Occupational and Environmental Medicine, Department of Laboratory Medicine, Lund University, Lund, Sweden; 2 Department of Dermatology, Skåne University Hospital, Lund, Sweden; 3 Dermatology, Department of Clinical Sciences, Lund University, Lund, Sweden; 4 Department of Occupational and Environmental Medicine, Region Skåne, Lund, Sweden; 5 Division of Orthopaedics, Clinical Sciences Lund, Lund University and Skåne University Hospital, Lund, Sweden; 6 Clinical Pharmacology, Pharmacy and Environmental Medicine, Institute of Public Health, University of Southern Denmark, Odense, Denmark

We appreciate the significant attention our recent publication [1] has gained. This heightened interest underscores important concerns regarding the public health effects of tattoo exposure and emphasizes the urgent need for a more comprehensive understanding of tattoo toxicity. Our findings add one piece to this emerging evidence. Moving the field forward will require research across disciplines, and we remain committed to supporting that development.

The main points raised in the letters by Faheem [2], Kluger [3], and McCarty et al. [4] relate to the possibility that our results could be artifacts arising from different types of biases, questions about outcome and exposure validity, (unstated) claims of causality, and limitations in the study design. These are all central concerns in observational research that need to be carefully addressed to obtain valid results.

In contrast to studies that added tattoo-related questions to long-standing cohort infrastructures, our study was designed from the outset to investigate the association between tattoo exposure and cutaneous melanoma. This allowed us to collect purpose-specific data tailored to accurately assess both exposure and relevant covariates. We put meticulous effort into designing a study that minimized the potential for bias and ensured the generation of robust and reliable results. We therefore welcome the opportunity to clarify the rationale behind our analytical approach and to contribute to a solid scientific foundation for future research in this emerging field.

## Selection bias

A challenge in case-control studies is to select controls that represent the target population. We used a nested case-control design, where the study participants were selected from the total underlying population. This is possible in the Nordic countries because of administrative registers with mandatory participation and implies nearly perfect overlap between the source population and the target population. Furthermore, by embedding the case–control sample within the complete underlying population, the controls accurately reflect the exposure distribution in the source population from which the cases arose.

Selection bias can arise in case–control studies if participation is conditioned on both exposure and outcome status. To minimize this risk, our questionnaire was deliberately broad in scope, and participants were not informed that the study concerned tattoos and cutaneous melanoma. Instead, the stated aim was to investigate general lifestyle factors and their potential associations with non-specific cancer and other diseases. The questionnaire covered a wide range of exposures – including tattoos, piercing, scarification, henna tattoos, hair-dyeing practices, various laser treatments, sun habits, tanning-bed use, smoking, snuff use, and alcohol consumption – making it highly unlikely that participation would be conditioned on any single exposure.

Ideally, access to individual-level data on non-participants would have allowed a formal quantitative bias analysis. As this was not possible for reasons of personal integrity, we instead evaluated the internal coherence of the data by examining the distribution of established melanoma risk factors. As expected, characteristics such as fair skin, childhood sunburns, and tanning-bed use were more common among cases than controls, supporting the validity of the data. In addition, the overall tattoo prevalence in our study was well in line with the published literature, further supporting that participation was not selectively influenced by tattoo status. Taken together, these factors indicate that selection into the study is unlikely to have introduced substantial bias into the observed association.

## Confounding

We applied an a priori Directed Acyclic Graph (DAG) informed by the published literature to identify potential confounders. We did not adjust for variables that did not meet the confounding criteria, i.e., that were not assumed to be related to both the exposure and the outcome. For this reason, we did not adjust for some established risk factors – such as familial history of melanoma – because in our DAG there is no causal path between familial history and tattoo exposure other than through socioeconomic characteristics, which were already accounted for. Adjustment for variables that do not lie on a backdoor path between exposure and outcome may lead to overadjustment bias, adding model complexity and noise without reducing confounding, thereby reducing statistical precision and potentially attenuating the effect estimates.

In contrast, strong predictors of the outcome that are not associated with exposure status can be included as precision variables. These variables do not block any backdoor paths but can reduce unexplained variability in the outcome, thereby increasing the precision of the estimated association. For this reason, we included strong risk factors with limited relevance for tattoo decisions - such as childhood sunburn - to improve precision without introducing bias. Factors with a familial component that may be related to exposure status, including Fitzpatrick skin type (genetic factor) and sun behaviour (shared environmental factor), were instead accounted for through the phenotypic risk index and the UV-exposure index.

We aggregated the different dimensions of UV exposure into a single composite index due to the substantial intercorrelation among the individual measures. Modelling a unified index, rather than including each variable separately, reduced multicollinearity while preserving the underlying exposure information. Moreover, composite measures are generally less susceptible to misclassification than individual self-reported variables, as integrating several correlated indicators diminishes the influence of noise arising from any single component.

Faheem [2] and McCarty et al. [4] raise concerns of residual confounding arising from insufficient adjustment of UV-exposure, the major risk factor for melanoma. There are indeed challenges associated with self-reported data on UV-exposure that may impact precision. To ensure reliable exposure data, we used a validated battery of questions for self-assessment of UV-exposure in Swedish settings, developed by cancer epidemiologists specifically for population-based research. Although not perfect, it is best-practice as personal exposure monitoring is not practically feasible in large-scale studies.

To evaluate the potential impact of residual confounding, the E-value [5] can be applied to quantify how strong an unmeasured confounder would need to be to account for the observed association between exposure and outcome. After adjustment for sex, age, household disposable income, marital status, educational attainment, UV-exposure index, phenotypic risk index, and smoking status, we observed an association between tattoo exposure and melanoma of RR = 1.29 (95% CI: 1.07–1.56). In our context, the E-value provides an estimate of the minimum strength of association that an unmeasured confounder would need to have with both tattoo exposure and melanoma, beyond these measured covariates, to fully explain the results. The E-value for the point estimate was 1.9, indicating that only a confounder associated with both tattoo status and melanoma by a risk ratio of at least 1.9 each could eliminate the observed association. The E-value for the lower confidence bound was 1.34, meaning that even to attenuate the estimate to include the null, an unmeasured confounder of this magnitude would be required.

Importantly, the interpretation of the E-value also encompasses residual confounding due to measurement error in the adjusted covariates: any mismeasurement in variables such as UV-exposure or phenotypic risk would have to exert an influence equivalent to that of an unmeasured confounder with the strength indicated by the E-value. Given the breadth of covariates adjusted for, and the fact that the remaining residual confounding from measurement error would need to be substantial to eliminate the association, it is unlikely that such bias alone accounts for our findings.

## Exposure assessment

We agree with Faheem [2] and McCarty et al. [4] that improved exposure assessment is needed in this field. However, epidemiologic research on rare outcomes necessarily involves a trade-off between sample size and the level of exposure detail that can realistically be obtained. Our study was therefore designed and powered around a dichotomous measure of tattoo exposure. Given the current lack of mechanistic understanding of tattoo toxicology, more granular exposure metrics cannot yet be defined in a biologically meaningful way.

At this stage of tattoo research, the primary epidemiologic objective is to determine whether any association exists between tattoo exposure and adverse health outcomes. More refined exposure assessment presupposes an evidence base that is still emerging. Meaningful exposure characterization would require understanding of the relative contributions of initial chemical exposure, degradation products formed over time, the particulate load itself, and the immunological consequences of persistent foreign bodies within the lymphatic system, as well as validated knowledge of pigment distribution across physiological compartments. As such mechanistic insights accumulate, future studies will be able to develop exposure metrics that better reflect the underlying biology. Until then, epidemiologic analyses must rely on feasible, reproducible exposure classifications that minimize misclassification while preserving statistical power.

In line with the current understanding of how tattoo pigments behave, we argue that tattoo exposure should not be viewed as a one-time intradermal event, contrary to what is suggested in the letter by Kluger [3]. Rather than constituting a single, localized exposure, it represents a dynamic, systemic exposure process. This broader understanding should inform the future direction of research in this field.

## Outcome assessment

The concern raised by Kluger [3] regarding the inclusion of melanocytic nevi with high-grade dysplasia reflects the ongoing challenges in distinguishing these lesions from melanoma in situ. In Sweden, these lesions are managed in the same way and are registered together in the National Cancer Register. Our grouping therefore reflects established clinical and registry practice. Taken together with the value of examining early melanocytic alterations, their inclusion in the precursor group is both reasonable and epidemiologically appropriate. To address this point transparently, we presented separate analyses for invasive melanoma, melanoma in situ, and the largest subtypes within each. While Kluger’s letter [3] highlights that the confidence interval for overall invasive melanoma marginally includes 1, it does not consider that the largest invasive subtype – superficial spreading melanoma – showed an association (RR 1.40, 95% CI 1.03–1.90). In addition, the melanocytic nevi subgroup, which includes high-grade dysplastic nevi, showed an elevated risk (IRR 1.39, 95% CI 1.05–1.85). These consistent patterns across clinically relevant subgroups indicate that our findings are not driven by how high-grade dysplastic nevi were classified but rather reflect a broader and coherent association.

In line with good epidemiologic practice, we followed a strict hypothesis-based study protocol to guide all analytical decisions and to avoid data-driven choices and the fallacies associated with post hoc testing. For example, we did not have an a priori hypothesis that localization would matter, given that we view immune disruption as a potential pathobiological mechanism. This interpretation is supported by recent experimental work indicating that tattooing can induce prolonged lymph-node inflammation and alter vaccine responses in mice [6]. Consequently, we did not conduct a sensitivity analysis restricted to individuals with tattoos and tumors at the same anatomical site, as this would have constituted a post hoc, data-driven approach. Likewise, we adhered to the study protocol and did not modify the primary outcome in response to the sensitivity analyses, including the analysis restricted to first-time melanomas. Here, we would like to emphasize that the results were consistent regardless of how cases were defined – whether we included only first-time melanomas, distinguished between in situ and invasive cases, varied the included covariates, or accounted for childhood burns. All analyses indicated a consistent association.

## Absence of dose-response relationship

The authors of the letters [2–4] raise concerns about the absence of an exposure–response relationship. Indeed, a distinct dose–response relationship – corresponding to the biological gradient criterion outlined by Bradford Hill [7] – can strengthen causal inference, yet its interpretation presupposes a well-defined and biologically meaningful exposure metric. Such metrics are not currently available in the context of tattoo toxicology. The internal dose is unlikely to be proportional to visible tattoo size, the exposure is systemic and dynamic rather than local, and misclassification of tattooed body surface area is likely non-differential. Such non-differential misclassification may also attenuate risk estimates in the highest exposure category, potentially explaining why risk increases appear strongest among individuals with smaller tattoos. Therefore, the absence of a dose-response pattern cannot be taken as evidence against an association at this early stage of the field.

McCarty et al. [4] suggest that the increased risk observed among individuals with small tattoos may reflect the inclusion of medical tattoos, given that these individuals may have an elevated melanoma risk due to their underlying medical history. While this is a reasonable consideration, it is unlikely to account for our findings. Individuals with medical tattoos constituted a very small proportion of our study population (10 cases and 15 controls), making it implausible that they materially influenced the observed association. Moreover, if this small subgroup had meaningfully biased the results, we would expect the effect to be unstable or sensitive to analytic choices, rather than confined to a single exposure category. The observed pattern is instead more consistent with exposure misclassification of tattoo size and with the broader challenges of defining a biologically meaningful exposure metric at this early stage of tattoo-related research.

## Causality

A recurring point in all letters [2–4] is the inability of our study to prove causality. We agree that establishing causality requires more than a single epidemiological study; it encompasses a combination of multiple cornerstones, including – but not limited to – reproducibility and plausible mechanistic pathways. As tattoo biology remains an emerging field, the available evidence is still limited, but the initial evidence is concerning. Or – as we state in the Conclusion – our data suggested that tattoos may be a risk factor for cutaneous melanoma, and these results now need to be verified by carefully designed epidemiologic studies before causality can be inferred.

## Study design considerations

We agree with Faheem [2] and McCarty et al. [4] that prospective cohorts with sufficient numbers of tattooed participants to achieve statistical power for rare outcomes – combined with objective exposure assessment informed by advances in tattoo toxicology, analytic characterization of ink components, and longitudinal biological sampling for effect biomarkers in different matrices – would represent the ideal study design. However, building such infrastructures would require extensive follow-up periods and substantial financial resources. Given the high prevalence of tattooing and the need to generate evidence without delay, this approach is not feasible at present.

In this context, population-based case-control studies remain an efficient and scientifically valid design. Although retrospective exposure assessment is not without limitations, our study leveraged all incident cases of cutaneous melanoma in the entire Swedish population aged 20–60 years in 2017 – approximately five million individuals – thereby ensuring a comprehensive source population and strong statistical power. This makes it a methodologically solid design for addressing the research question with the data currently available.
